# Supplementary material for: Multi‐dataset identification of innovative feature genes and molecular mechanisms in keratoconus
Source: J Cell Mol Med. 2024 Sep 19;28(18):e70079. doi: 10.1111/jcmm.70079 (PMC11412914; doi:10.1111/jcmm.70079)
Supplement: Supplementary file 2 — Material S2. [file JCMM-28-e70079-s002.zip › 7.GSEA analysis/1.ARL11/KEGG_MELANOGENESIS.html]

Details for gene set KEGG\_MELANOGENESIS[GSEA]

|  || Dataset | ARL11.group.cls#h\_versus\_l |
| Phenotype | group.cls#h\_versus\_l |
| Upregulated in class | l |
| GeneSet | KEGG\_MELANOGENESIS |
| Enrichment Score (ES) | -0.2763925 |
| Normalized Enrichment Score (NES) | -1.0676086 |
| Nominal p-value | 0.35632184 |
| FDR q-value | 1.0 |
| FWER p-Value | 0.998 |
Table: GSEA Results Summary

  

Fig 1: Enrichment plot: KEGG\_MELANOGENESIS      
 Profile of the Running ES Score & Positions of GeneSet Members on the Rank Ordered List

  

| SYMBOL | TITLE | RANK IN GENE LIST | RANK METRIC SCORE | RUNNING ES | CORE ENRICHMENT || 1 | PLCB2 | na | 134 | 0.619 | 0.0544 | No |
| 2 | MC1R | na | 1861 | 0.306 | 0.0334 | No |
| 3 | CALML6 | na | 3001 | 0.240 | 0.0231 | No |
| 4 | WNT5A | na | 3234 | 0.230 | 0.0381 | No |
| 5 | CAMK2B | na | 3930 | 0.203 | 0.0371 | No |
| 6 | ADCY3 | na | 5276 | 0.158 | 0.0132 | No |
| 7 | WNT9A | na | 5395 | 0.155 | 0.0243 | No |
| 8 | ADCY2 | na | 5416 | 0.154 | 0.0382 | No |
| 9 | ADCY9 | na | 5439 | 0.153 | 0.0520 | No |
| 10 | ADCY7 | na | 5559 | 0.149 | 0.0626 | No |
| 11 | FZD4 | na | 5857 | 0.141 | 0.0673 | No |
| 12 | WNT10B | na | 6703 | 0.119 | 0.0541 | No |
| 13 | CAMK2A | na | 6879 | 0.114 | 0.0598 | No |
| 14 | CREBBP | na | 7701 | 0.098 | 0.0453 | No |
| 15 | ADCY1 | na | 7855 | 0.095 | 0.0498 | No |
| 16 | GNAI3 | na | 7891 | 0.095 | 0.0578 | No |
| 17 | FZD1 | na | 8051 | 0.091 | 0.0618 | No |
| 18 | WNT3A | na | 8640 | 0.080 | 0.0524 | No |
| 19 | PRKX | na | 9610 | 0.066 | 0.0305 | No |
| 20 | PLCB3 | na | 10228 | 0.057 | 0.0181 | No |
| 21 | ADCY4 | na | 10290 | 0.056 | 0.0217 | No |
| 22 | ADCY6 | na | 10456 | 0.054 | 0.0220 | No |
| 23 | PRKCA | na | 10617 | 0.052 | 0.0223 | No |
| 24 | KRAS | na | 10630 | 0.052 | 0.0269 | No |
| 25 | TCF7L2 | na | 10815 | 0.050 | 0.0262 | No |
| 26 | KITLG | na | 11152 | 0.046 | 0.0209 | No |
| 27 | CAMK2G | na | 11259 | 0.045 | 0.0220 | No |
| 28 | FZD5 | na | 11914 | 0.037 | 0.0066 | No |
| 29 | FZD6 | na | 12404 | 0.032 | -0.0045 | No |
| 30 | WNT8A | na | 12464 | 0.032 | -0.0032 | No |
| 31 | FZD3 | na | 12567 | 0.031 | -0.0033 | No |
| 32 | WNT1 | na | 12803 | 0.029 | -0.0073 | No |
| 33 | CREB3L1 | na | 12911 | 0.028 | -0.0078 | No |
| 34 | GNAI2 | na | 13045 | 0.027 | -0.0092 | No |
| 35 | CALM2 | na | 14606 | 0.014 | -0.0529 | No |
| 36 | CTNNB1 | na | 14653 | 0.014 | -0.0529 | No |
| 37 | POMC | na | 15660 | 0.006 | -0.0814 | No |
| 38 | KIT | na | 16102 | 0.003 | -0.0938 | No |
| 39 | WNT3 | na | 16162 | 0.003 | -0.0953 | No |
| 40 | GNAS | na | 16257 | 0.002 | -0.0978 | No |
| 41 | ADCY5 | na | 16504 | 0.000 | -0.1049 | No |
| 42 | WNT10A | na | 16653 | -0.001 | -0.1091 | No |
| 43 | MAP2K2 | na | 16997 | -0.003 | -0.1187 | No |
| 44 | CALML5 | na | 17116 | -0.004 | -0.1217 | No |
| 45 | CREB3L4 | na | 17310 | -0.005 | -0.1268 | No |
| 46 | DCT | na | 17406 | -0.006 | -0.1290 | No |
| 47 | PRKCG | na | 17545 | -0.007 | -0.1323 | No |
| 48 | GNAQ | na | 17828 | -0.009 | -0.1396 | No |
| 49 | PLCB4 | na | 18133 | -0.011 | -0.1473 | No |
| 50 | TCF7 | na | 18185 | -0.012 | -0.1476 | No |
| 51 | HRAS | na | 18435 | -0.013 | -0.1536 | No |
| 52 | WNT4 | na | 18621 | -0.014 | -0.1576 | No |
| 53 | CALML3 | na | 18646 | -0.015 | -0.1569 | No |
| 54 | CALM1 | na | 18762 | -0.015 | -0.1588 | No |
| 55 | TYRP1 | na | 18888 | -0.016 | -0.1608 | No |
| 56 | PRKACB | na | 18969 | -0.017 | -0.1616 | No |
| 57 | DVL3 | na | 19519 | -0.021 | -0.1755 | No |
| 58 | WNT5B | na | 19914 | -0.023 | -0.1847 | No |
| 59 | FZD10 | na | 20030 | -0.024 | -0.1858 | No |
| 60 | GSK3B | na | 20728 | -0.029 | -0.2031 | No |
| 61 | CREB3 | na | 20880 | -0.030 | -0.2047 | No |
| 62 | DVL2 | na | 22249 | -0.042 | -0.2402 | No |
| 63 | WNT2 | na | 22576 | -0.046 | -0.2453 | No |
| 64 | MAPK3 | na | 22614 | -0.046 | -0.2420 | No |
| 65 | CALM3 | na | 22971 | -0.050 | -0.2476 | No |
| 66 | CREB3L2 | na | 23209 | -0.052 | -0.2495 | No |
| 67 | ASIP | na | 23255 | -0.053 | -0.2458 | No |
| 68 | RAF1 | na | 23336 | -0.054 | -0.2431 | No |
| 69 | EDNRB | na | 23504 | -0.056 | -0.2426 | No |
| 70 | PRKACA | na | 23665 | -0.058 | -0.2418 | No |
| 71 | DVL1 | na | 23820 | -0.060 | -0.2406 | No |
| 72 | EP300 | na | 23862 | -0.060 | -0.2361 | No |
| 73 | TCF7L1 | na | 24530 | -0.070 | -0.2488 | No |
| 74 | MAPK1 | na | 24626 | -0.071 | -0.2448 | No |
| 75 | FZD9 | na | 25720 | -0.086 | -0.2683 | Yes |
| 76 | PRKACG | na | 25752 | -0.087 | -0.2611 | Yes |
| 77 | ADCY8 | na | 25957 | -0.090 | -0.2585 | Yes |
| 78 | TYR | na | 26252 | -0.094 | -0.2581 | Yes |
| 79 | EDN1 | na | 26499 | -0.099 | -0.2560 | Yes |
| 80 | WNT7A | na | 26706 | -0.103 | -0.2523 | Yes |
| 81 | CREB1 | na | 27227 | -0.113 | -0.2566 | Yes |
| 82 | GNAI1 | na | 27299 | -0.115 | -0.2479 | Yes |
| 83 | FZD8 | na | 27395 | -0.117 | -0.2397 | Yes |
| 84 | WNT7B | na | 27789 | -0.125 | -0.2392 | Yes |
| 85 | NRAS | na | 27790 | -0.126 | -0.2274 | Yes |
| 86 | CREB3L3 | na | 27885 | -0.128 | -0.2181 | Yes |
| 87 | WNT16 | na | 28062 | -0.132 | -0.2107 | Yes |
| 88 | FZD2 | na | 28464 | -0.142 | -0.2089 | Yes |
| 89 | GNAO1 | na | 28977 | -0.156 | -0.2090 | Yes |
| 90 | WNT6 | na | 29533 | -0.173 | -0.2087 | Yes |
| 91 | FZD7 | na | 30073 | -0.190 | -0.2064 | Yes |
| 92 | WNT9B | na | 30689 | -0.211 | -0.2043 | Yes |
| 93 | MAP2K1 | na | 31070 | -0.223 | -0.1943 | Yes |
| 94 | WNT8B | na | 31805 | -0.253 | -0.1918 | Yes |
| 95 | CAMK2D | na | 32170 | -0.268 | -0.1771 | Yes |
| 96 | PLCB1 | na | 32251 | -0.272 | -0.1538 | Yes |
| 97 | LEF1 | na | 32575 | -0.292 | -0.1356 | Yes |
| 98 | PRKCB | na | 33863 | -0.399 | -0.1352 | Yes |
| 99 | WNT11 | na | 33983 | -0.417 | -0.0993 | Yes |
| 100 | MITF | na | 34468 | -0.521 | -0.0643 | Yes |
| 101 | WNT2B | na | 34700 | -0.762 | 0.0008 | Yes |
Table: GSEA details [plain text format]

  

Fig 2: KEGG\_MELANOGENESIS      
 Blue-Pink O' Gram in the Space of the Analyzed GeneSet

  

Fig 3: KEGG\_MELANOGENESIS: Random ES distribution      
 Gene set null distribution of ES for **KEGG\_MELANOGENESIS**
